# Characterization of Seminal Plasma Extracellular Vesicle MicroRNAs and Their Association with Boar Semen Quality During the Summer Season

**DOI:** 10.3390/ijms27104548

**Published:** 2026-05-19

**Authors:** Notsile H. Dlamini, Serge L. Kameni, Shengfa F. Liao, Jean M. Feugang

**Affiliations:** Department of Animal and Dairy Sciences, Mississippi State University, Mississippi State, MS 39762, USA; nhd30@msstate.edu (N.H.D.); sergekl@outlook.com (S.L.K.); s.liao@msstate.edu (S.F.L.)

**Keywords:** boar, seminal plasma, extracellular vesicles, semen quality, microRNAs

## Abstract

Boar fertility is negatively affected by subfertility and elevated temperatures, which alter seminal plasma (SP) composition and reduce semen quality. Extracellular vesicles (EVs) in SP transfer microRNAs (miRNAs) to sperm and may influence sperm function. This study aimed to identify SP-EV microRNAs associated with differences in boar semen quality during the summer season. Semen collected from Duroc boars was evaluated and classified as Passed (≥70%) or Failed (<70%) based on sperm quality parameters. SP-EVs were isolated and characterized, and small RNA sequencing was performed to profile miRNA content. SP-EVs ranged from 90 to 200 nm, with concentrations of 4.33 × 10^10^ particles/mL in the Passed group and 1.85 × 10^11^ particles/mL in the Failed group. Western blotting confirmed the presence of EV surface markers CD9, CD63, and CD81. A total of 446 unique miRNAs were identified, with 28 downregulated and two upregulated miRNAs in Passed compared with Failed SP-EVs. Additionally, functional enrichment analysis revealed that target genes of upregulated miRNAs were involved in sperm-related biological processes and PI3K-Akt, regulation of actin cytoskeleton, and ErbB signaling pathways. These findings demonstrate that SP-EV miRNAs reflect physiological responses to changes in environmental conditions and may contribute to the regulation of boar semen quality.

## 1. Introduction

In the commercial pig industry, the accurate evaluation and selection of high-fertility boars are critical for optimizing conception rates and increasing profitability. Traditionally, fertility assessment in male livestock has relied heavily on conventional semen quality parameters, including computer-assisted sperm analysis (CASA). CASA is a widely used and reliable method for assessing sperm motility, morphology, and kinetic parameters in both field and laboratory settings because it can precisely track sperm motion velocities [1,2]. Another approach is flow-cytometry-based semen analysis, which is used to evaluate sperm viability, DNA content, acrosomal integrity, mitochondrial membrane potential, and other determinants of sperm quality [3,4]. Currently, CASA is the simpler and more affordable option; however, recent studies suggest that these diagnostic methods exhibit limited predictive accuracy for fertility and reproductive performance in boars, thereby constraining the overall effectiveness of artificial insemination (AI) programs [5,6].

Suboptimal fertility affects semen quality and contributes to reduced reproductive performance [7,8]. A significant challenge in addressing this condition is the lack of reliable, molecular-based diagnostic tools. Current approaches are hindered by animal heterogeneity and sampling variability, often leading to inconclusive outcomes. Commercial farms often pool semen from multiple boars, including those with lower sperm quality, to increase total sperm count per insemination dose. This management practice, however, tends to mask subfertile boars, making it difficult to identify and remove them from the breeding herd [5]. Hence, there is a need to identify specific molecular markers to support both the accurate selection of high-fertility males and the development of targeted therapeutic interventions.

Semen quality is affected by various factors, including seasonality and individual variability. Elevated ambient temperatures during the summer months have been shown to impair testicular function and alter the secretory activity of accessory sex glands, ultimately compromising semen quality [9,10]. Although advancements such as CASA have improved semen evaluation, the lack of dependable predictive biosignatures for semen quality remains a significant limitation, highlighting the need for further research and innovation [8].

Seminal plasma (SP), the non-cellular component of semen, has emerged as a key focus in male reproductive biology, particularly through advanced omics approaches such as transcriptomics, metabolomics, and proteomics [11,12,13,14]. Seminal plasma is a complex fluid comprising diverse secretions from the testes, epididymis, and accessory sex glands that play a crucial role in sperm metabolism, survival, and function [15,16,17]. Among its components, extracellular vesicles (EVs) have garnered attention for their role in intercellular communication. Extracellular vesicles are small, nanosized, membrane-bound vesicles, approximately 30–120 nm, released by various cell types that carry diverse biomolecules, including proteins, lipids, and nucleic acids (mRNAs, microRNAs, DNA) to recipient cells for cell-to-cell communication [18,19,20,21,22]. Due to their ability to transport molecular cargo that reflects their cell of origin, EVs are promising candidates for biological signatures.

Numerous studies have highlighted the pivotal role of seminal plasma extracellular vesicles (SP-EVs) in modulating different sperm processes, such as sperm capacitation, motility, and acrosome reaction, by fusing with the sperm membrane to transfer proteins and small RNAs [23,24,25,26]. Notably, non-coding RNAs, particularly microRNAs (miRNAs), are emerging as key regulators of gene expression through transcriptional and post-transcriptional mechanisms [22,24,27]. MiRNAs are single-stranded, composed of 22–24 nucleotides, that regulate numerous genes associated with reproductive processes, including germ cell development, sperm maturation, capacitation, motility, and fertilization [28,29]. EV-derived miRNAs have the potential to serve as valuable biomarkers for diagnosing and improving genetic resilience to environmental stressors, such as heat. Heat stress occurs when the body struggles to dissipate heat effectively, leading to seasonal infertility. Particularly in boar studs, elevated temperatures can adversely affect sperm quality during and after spermatogenesis [30], resulting in increased semen rejection rates during the summer [31], with total motility falling below 70% and morphological defects exceeding 30% [31,32]. Yet, the influence of miRNAs derived from SP-EVs on sperm function under heat stress remains poorly understood.

In a previous study, we profiled miRNAs in SP-EVs collected from boar semen during winter, revealing a distinct profile associated with varying semen quality [33]. In this follow-up study, we hypothesize that miRNAs in boar SP-EVs contribute to the variations in semen quality observed during the summer months. Our objective is to analyze the miRNA content of boar SP-EVs in relation to changes in semen quality throughout the summer and to explore the biological functions of differentially expressed miRNAs in sperm function. We expect our findings to identify miRNAs relevant to seasonal variation.

## 2. Results

### 2.1. Semen Quality Analysis

**Figure 1:**
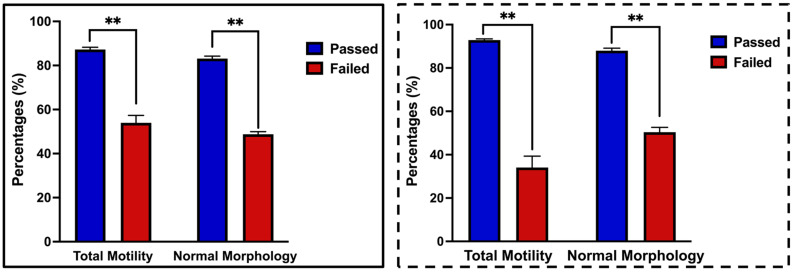
Total motility and normal morphology of collected semen. The solid box represents semen quality parameters for the overall samples (*n* = 38 Passed and *n* = 45 Failed), while the dashed box represents samples selected for miRNA analysis (*n* = 5 Passed and *n* = 5 Failed). Data are shown as mean ± SEM, and asterisks (**) indicate significant differences at *p* < 0.001.

In Figure 1, the solid box represents the overall averages for total motility (87.2 ± 1.0% vs. 54.0 ± 3.4%) and normal morphology (86.3 ± 1.1% vs. 48.8 ± 1.2%), both of which were significantly higher (*p* < 0.001) in the Passed group (*n* = 38) than in the Failed group (*n* = 45). The dashed box shows the characteristics of the samples selected for miRNA analysis, which exhibited similar trends, with significant differences in total motility (92.9 ± 0.6% vs. 34.1 ± 5.3%) and normal morphology (88.0 ± 1.2% vs. 50.4 ± 2.2%) between Passed (*n* = 5) and Failed (*n* = 5) samples, respectively.

### 2.2. SP-EVs Characteristics

**Figure 2:**
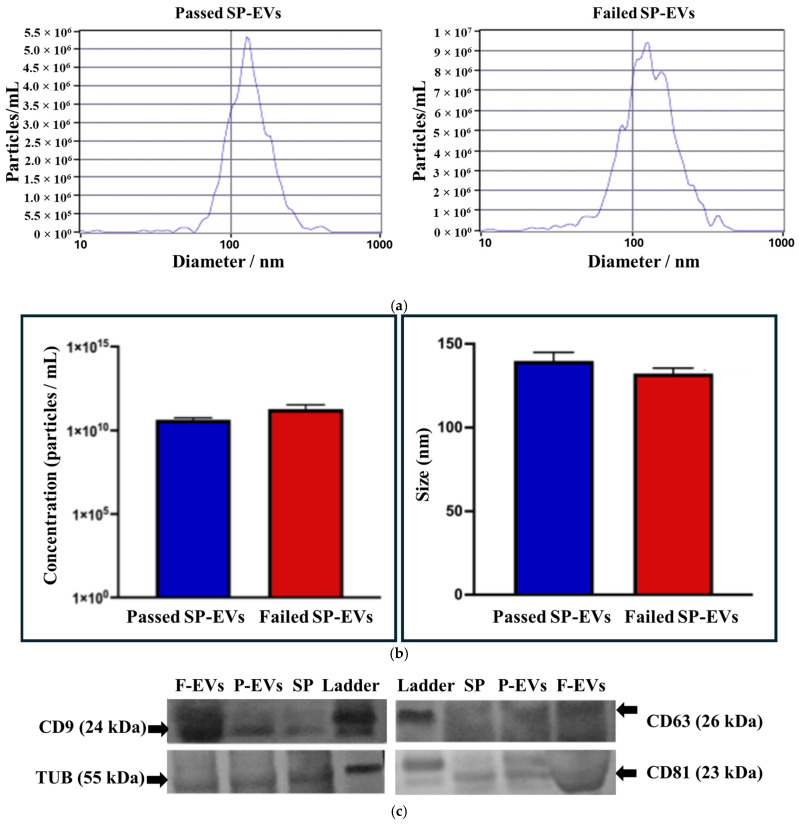
Nanoparticle tracking analysis (NTA) and Western immunoblotting characterization of Passed and Failed SP-EVs. Figures are representative particle concentration and size distribution profiles (**a**), average data (±SD) of three analytical replicates of Passed and Failed SP-EVs (**b**), and blotting detecting CD9, CD63, and CD81 as markers of SP-EVs in Passed (P-EVs) and Failed (F-EVs) samples (**c**). Boar seminal plasma (SP) was loaded as the loading control, while detection of the cytoplasmic marker Tubulin with an antibody served as an internal control.

Figure 2a shows the NTA determination of SP-EVs concentration (particles/mL) and size distribution (hydrodynamic diameter, nm), with the average values for Passed samples (4.33 × 10^10^ particles/mL and 139.8 nm) and Failed samples (1.85 × 10^11^ particles/mL and 132.2 nm) indicated in Figure 2b. There was no significant difference in particle concentration and size distribution between Passed and Failed SP-EVs (*p* > 0.05). Western blotting confirmed the presence of EV protein markers, including CD9, CD63, and CD81, and the cytoplasmic marker β-Tubulin was strongly detected in seminal plasma (Figure 2c).

### 2.3. MicroRNA Profiling of Passed and Failed SP-EVs

**Figure 3:**
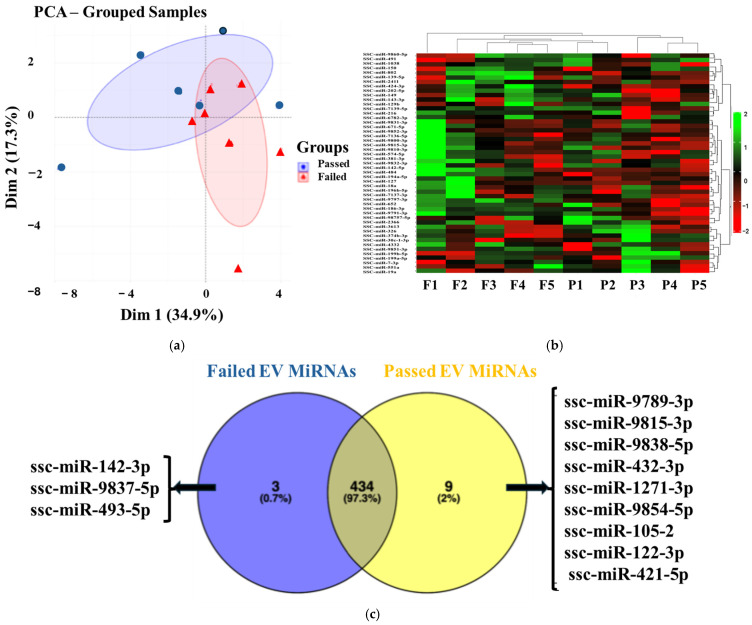
MicroRNA profiling of SP-EVs of Passed and Failed boar seminal plasma. (**a**) Principal component analysis of grouped samples (PCA) with Dim (PC) 1 and Dim (PC) 2 explaining 52.2% of the total variance in semen quality between the SP-EV groups. (**b**) The heatmap represents the top 50 variable miRNAs between Failed (F; *n* = 5) and Passed (P; *n* = 5) SP-EV samples. Downregulated (red) and upregulated (green) miRNAs in the Passed group are shown in red and green, respectively. Unchanged miRNA expression is shown in black. (**c**) The Venn diagram shows common (97.3%) and unique (2.7%) miRNAs expressed between Passed and Failed SP-EVs.

Small RNA-seq analysis revealed an average sequence length of 51 bp across all samples, which showed high-quality scores. Of the total reads, 257,654,373 (92.2%) were successfully mapped to the *Sus scrofa* genome, enabling the identification of various RNA biotypes, including tRNA, miRNA, ncRNA, snRNA, snoRNA, and piRNA. An average of 0.02% of the mapped reads were annotated to miRNAs from the miRbase database. Principal component analysis (PCA) and heatmap clustering revealed a clear clustering of biological replicates within the Failed SP-EV group, with lower variability than in the Passed SP-EV group (Figure 3a,b). In contrast, four biological replicates from the Passed SP-EV group exhibited greater variability relative to those in the Failed SP-EV group. Principal component analysis demonstrated that PC1 and PC2 explained 34.9% and 17.3% of the total variance in semen quality between the SP-EV groups, respectively. The separation of samples along PC1 suggests that semen quality is the dominant source of variability in the dataset, while PC2 captures additional heterogeneity that may reflect inter-individual variation.

A total of 443 and 437 miRNAs were detected with ≥5 raw reads in the Passed and Failed SP-EV groups, respectively. Of these, 446 miRNAs were unique, and 434 miRNAs (97.3%) were expressed in both the Passed and Failed SP-EV groups. Nine miRNAs (2%) were specific to the Passed group (ssc-miR-9789-3p, ssc-miR-9815-3p, ssc-miR-9838-5p, ssc-miR-432-3p, ssc-miR-1271-3p, ssc-miR-9854-5p, ssc-miR-105-2, ssc-miR-122-3p, and ssc-miR-421-5p), whereas three miRNAs (0.7%; ssc-miR-142, ssc-miR-9837-5p, and ssc-miR-493-5p) were specific to the Failed group (Figure 3c).

### 2.4. Identification of Differentially Expressed miRNAs Between Passed vs. Failed SP-EVs

**Figure 4:**
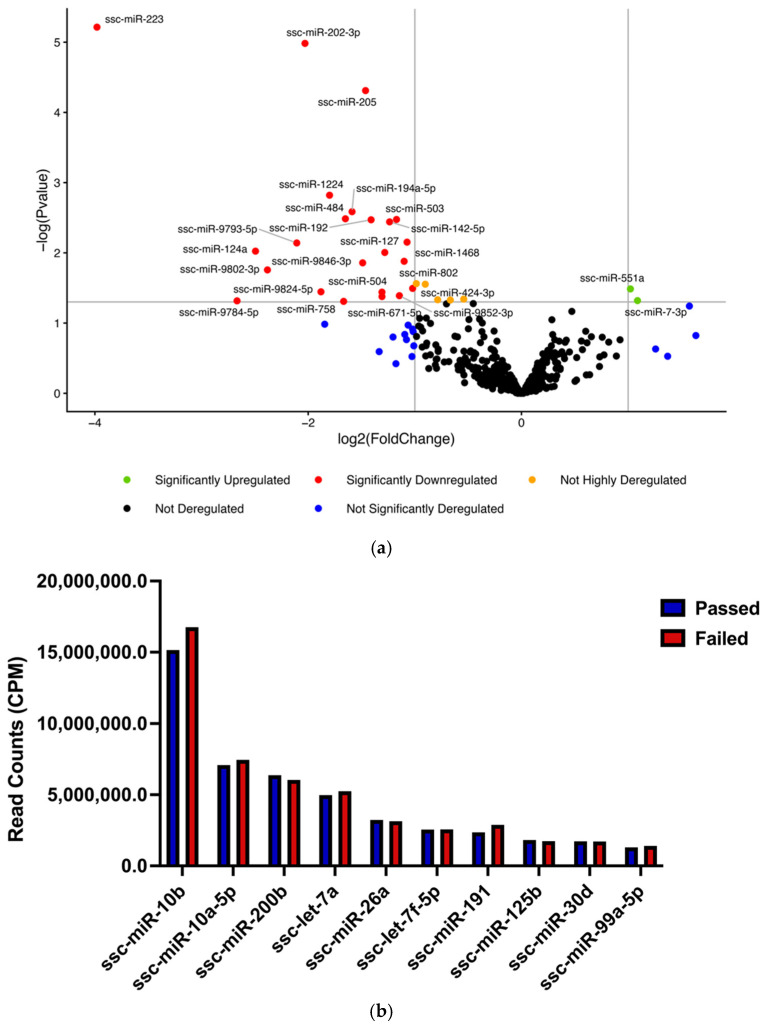
Differential expression analysis and top 10 most abundant miRNAs in Passed and Failed SP-EVs. (**a**) Volcano plot of differentially expressed miRNAs (DEMs: *n* = 30) between Passed and Failed SP-EV miRNAs. Each point in the volcano plot represents a miRNA. Red dots represent downregulated DEMs in the Passed group; green dots represent upregulated DEMs in the Passed group; blue dots represent miRNAs not significantly deregulated, and black dots represent miRNAs not deregulated. (**b**) The top 10 most abundant miRNAs in Passed and Failed SP-EVs.

The differential expression of SP-EV miRNAs between the Passed and Failed groups was assessed using a significance threshold of fold change (FC) > 1.5 or <0.7 and *p* < 0.05. This analysis identified 30 differentially expressed miRNAs (DEMs), including 28 that were downregulated and 2 (ssc-miR-551a and ssc-miR-7-3p) that were upregulated in the Passed SP-EV group (Table 1; Figure 4a). Notably, ssc-miR-223, ssc-miR-202-3p, and ssc-miR-205 were strongly downregulated according to FDR analysis. None of the DEMs identified were among the top 10 most abundant miRNAs in either group (Figure 4b).

### 2.5. Target Gene Prediction and Functional Enrichment Analysis of Differentially Expressed miRNAs

**Figure 5:**
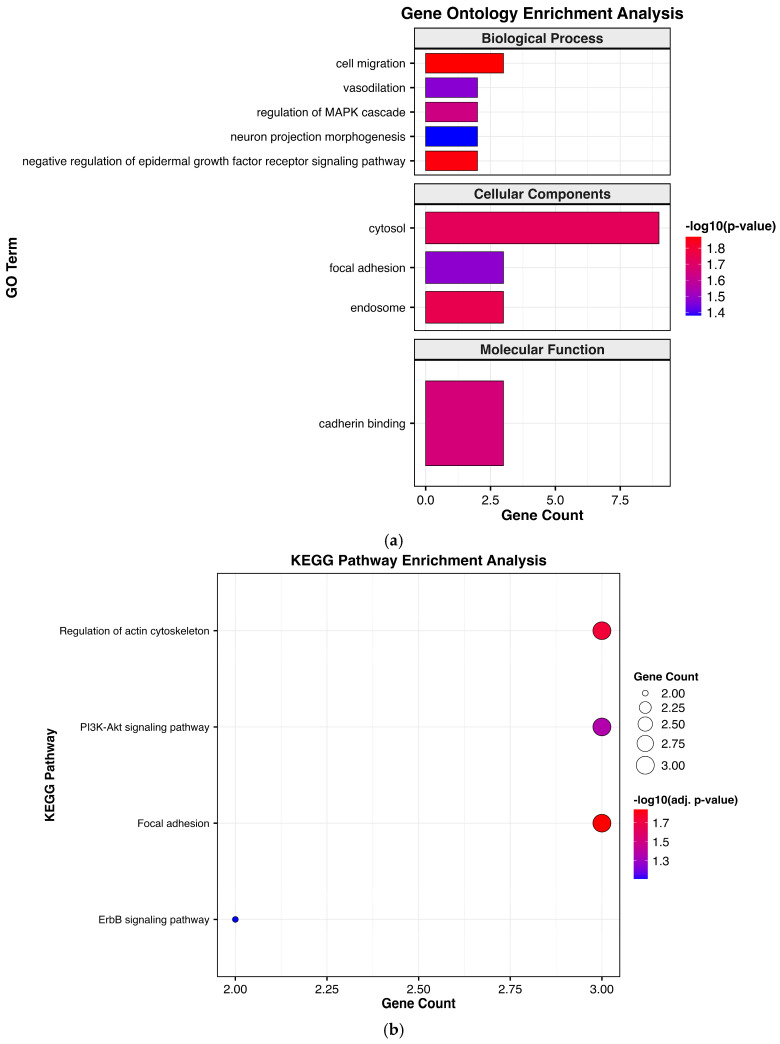
Functional enrichment analysis of the target genes of upregulated miRNAs in the Passed SP-EV group. (**a**) GO and (**b**) KEGG enrichment analysis of upregulated miRNAs in Passed SP-EVs.

Target gene prediction for DEMs was performed by aligning them with homologs human miRNAs using TargetScan and miRDB software. A total of 23 DEMs were successfully matched, collectively targeting 609 genes that were regulated by both up- and down-regulated miRNAs. These target genes were subsequently subjected to GO and KEGG enrichment analyses to better understand their potential regulatory roles. GO enrichment analysis of upregulated miRNAs in the Passed SP-EVs were associated with biological processes and cellular components, including cell migration, regulation of the MAPK cascade, vasodilation, cytosol, focal adhesion, endosome, and cadherin binding (Figure 5a). KEGG pathway enrichment analysis demonstrated that these miRNAs were involved in regulating the actin cytoskeleton, PI3K-Akt, focal adhesion, and ErbB signaling pathways (Figure 5b).

**Figure 6:**
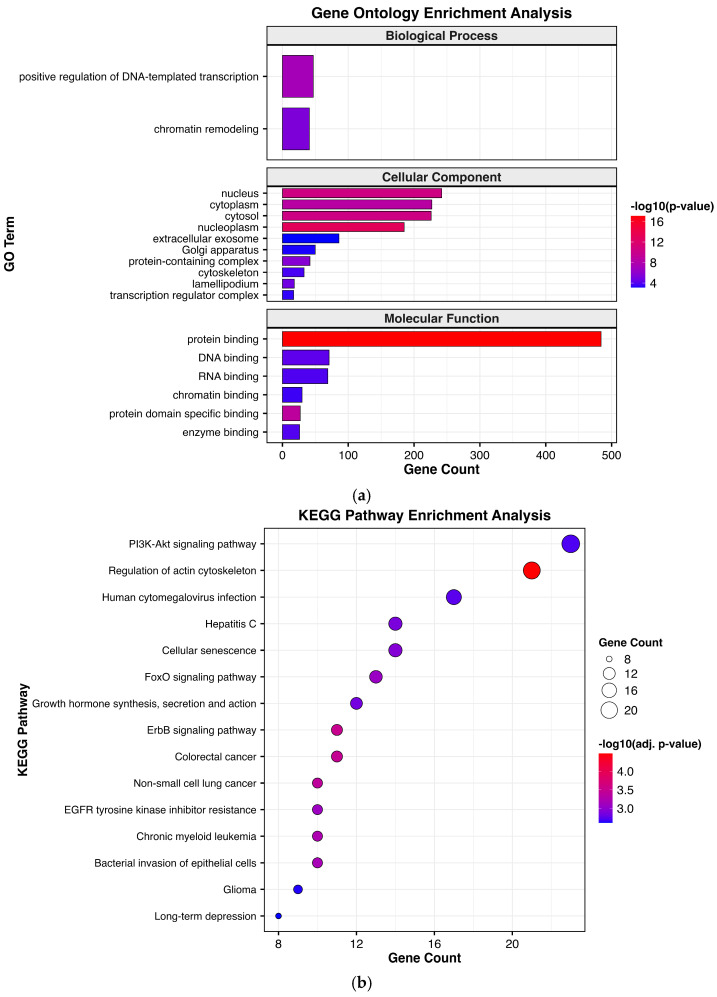
Functional enrichment analysis of the target genes of upregulated miRNAs in the Failed SP-EV group. (**a**) GO and (**b**) KEGG enrichment analysis of upregulated miRNAs in Failed SP-EVs.

In contrast, target genes of DEMs upregulated in Failed SP-EVs were mainly involved with GO terms related to the positive regulation of DNA-templated transcription, chromatin remodeling, and cellular components, including the nucleus, cytoplasm, cytosol, nucleoplasm, extracellular exosomes, Golgi apparatus, protein-containing complexes, cytoskeleton, lamellipodium, and transcription regulator complexes. These genes were also linked to molecular functions such as protein binding, DNA binding, RNA binding, chromatin binding, protein-domain-specific binding, and enzyme binding (Figure 6a). KEGG pathway analysis further revealed significant enrichment in multiple signaling pathways, including PI3K-Akt signaling, regulation of the actin cytoskeleton, cellular senescence, FoxO signaling, ErbB signaling, and EGFR tyrosine kinase inhibitor resistance (Figure 6b).

### 2.6. Validation of DEMs Using RT-qPCR

**Figure 7:**
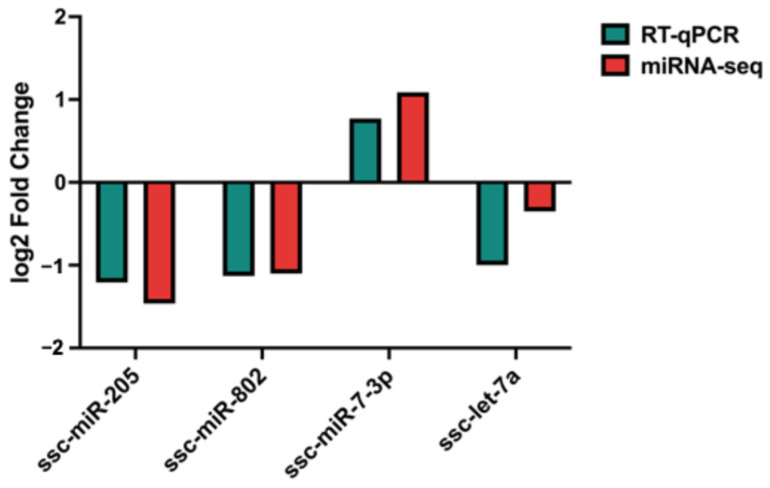
Validation of miRNA-sequencing data using RT-qPCR. The fold change was determined using the 2^−ΔΔCt^ method.

To assess the accuracy of the miRNA sequencing results, four differentially expressed miRNAs, namely ssc-miR-205, ssc-miR-802, ssc-miR-7-3p, and ssc-let-7a, were selected for validation through RT-qPCR based on their differential expression patterns and abundance detected in SP-EV groups. The results were expressed as log2 fold change (log2FC), and all miRNAs showed decreased expression in Passed relative to Failed SP-EV samples, with ssc-miR-7-3p exhibiting the greatest upregulation (log2FC = 0.771) (Figure 7). These findings were consistent with sequencing results, indicating that miRNA sequencing analysis was accurate. There were no significant differences in the expression levels of the selected miRNAs between Passed and Failed SP-EVs (*p* > 0.05).

## 3. Discussion

Seasonal heat stress is a well-recognized environmental factor contributing to reduced semen quality and fertility in boars, particularly during the summer months, when elevated ambient temperatures disrupt spermatogenesis, sperm maturation, and seminal plasma composition, ultimately affecting the profitability of commercial pig farms [34,35,36]. Despite extensive characterization of boar semen, the molecular mechanisms underlying summer-associated fertility decline in commercial boars remain incompletely understood. Boar seminal plasma (SP) contains a wide variety of organic and inorganic biochemical components, including extracellular vesicles (EVs), which play key roles in regulating sperm maturation, motility, capacitation, acrosome reaction, and sperm–zona pellucida binding [24,37,38].

Several studies have reported that sperm motility is associated with differences in miRNA expression in SP-EVs [26,33,39], supporting the potential of EV-associated miRNAs as molecular markers of boar sperm quality. In this study, we employed high-throughput sequencing to investigate miRNA profiles in high- and low-semen-quality samples from Duroc boars during the summer season. The presence of EVs in boar SP was validated using nanoparticle tracking analysis and western immunoblotting in accordance with the MISEV 2023 guidelines [40]. Our results demonstrated that boar SP contains a phenotypically heterogeneous population of EVs with particle sizes ranging from 90 to 200 nm and an average concentration of 4.33 × 10^10^ particles/mL for Passed SP-EVs whereas Failed SP-EVs were 1.85 × 10^11^ particles/mL. The higher SP-EV concentration observed in Failed samples likely reflects a stressed or dysregulated seminal environment and may contribute to impaired sperm function [41]. Western immunoblotting further confirmed the presence of EV surface markers CD9, CD63, and CD81, consistent with previous reports [24,25,33].

Small RNA sequencing revealed distinct EV-miRNA expression profiles between seminal plasma samples with acceptable (Passed) and poor (Failed) quality. A total of 446 unique miRNAs were detected, with 443 in Passed and 437 in Failed samples, including 434 shared and 12 group-specific miRNAs. The twelve specific miRNAs included three in Failed SP-EVs (miR-142-3p, miR-9837-3p, and miR-493-5p) and nine in Passed SP-EVs (miR-9789-3p, miR-9815-3p, miR-9838-5p, miR-432-3p, miR-1271-3p, miR-9854-5p, miR-105-2p, miR-122-3p, and miR-421-5p). Conflicting reports exist regarding the effects of elevated miR-142-3p levels in semen. Its presence in semen exosomes has been proposed as a biomarker for prostate cancer (PCa), whereas its detection in sperm has been associated with miscarriage risk in humans [42], and high motility in boars and bulls [43,44], and transgenerational epigenetic inheritance in rat models [45]. Interestingly, a recent study reported that miR-142-3p expression does not affect sperm motility [42]. Although miR-142-3p can bind to Akt3 and induce oxidative stress and DNA damage by increasing intracellular reactive oxygen species, its high activity may also inactivate the DNA damage response pathway [46]. Therefore, the presence of miR-142-3p in Failed samples may reflect an attempt by spermatozoa to counteract oxidative stress during summer.

MiR-493-5p is another miRNA uniquely detected in Failed SP-EV samples. In humans, this miRNA has been identified as a potential indicator of male infertility and is associated with poor sperm quality, particularly teratozoospermia (abnormal sperm morphology), due to its higher expression in teratozoospermic compared with normozoospermic individuals [47]. Additionally, miR-493-5p has been reported among testicular miRNAs associated with Sertoli cell-only syndrome, which results in abnormal sperm counts [48]. However, this finding contrasts with our previous report showing upregulation of miR-493-5p in SP-EVs from Passed samples during winter (log2FC = 2.846) [33]. Thus, we attribute this discrepancy to potential seasonal variation, which warrants further investigation to clarify the role of miR-493-5p in boar semen. Conversely, several miRNAs identified in Passed samples (e.g., miR-432-3p and miR-122-3p) have been positively correlated with sperm quality parameters such as motility, morphology, and concentration, as well as with male age and fertility status across multiple species [48,49].

Among the shared miRNAs, 30 were differentially expressed between SP-EV groups. Expression analysis revealed two significantly upregulated miRNAs (ssc-miR-551a and ssc-miR-7-3p) and 28 downregulated miRNAs in Passed SP-EVs. Similar patterns have been reported [26], who observed increased miRNA expression in low-motility semen exosomes in Landrace and Yorkshire boars. Likewise, Wang et al. [50] reported that numerous differentially expressed miRNAs (DEMs) were overexpressed in oligozoospermic patients compared with normal controls. Notably, three downregulated DEMs in the Passed group, ssc-miR-205, ssc-miR-802, and ssc-miR-9846-3p, were detected in both winter and summer boar SP-EVs with a similar consistent downregulation pattern, suggesting potential seasonal stability of these miRNAs [33,50].

Several DEMs identified in this study have previously been implicated in male infertility and spermatogenic disorders, processes known to be exacerbated by elevated temperatures. Ding et al. [51] reported that ssc-miR-1468 and ssc-miR-196a, which were downregulated in our study, were also differentially expressed between high- and low-motility SP-EVs. Additionally, ssc-miR-503, another downregulated miRNA, was previously identified in low-freezability boar SP-EVs and is predicted to regulate genes involved in cell cycle progression, differentiation, proliferation, and survival during male germ cell development [52]. Interestingly, one of the upregulated miRNAs, ssc-miR-551a, was also identified in exosomes from Landrace and Yorkshire boar semen, suggesting a regulatory role in enhancing sperm motility [26]. There is currently no direct evidence that ssc-miR-551a alone is a validated indicator of boar semen quality, but its expression pattern suggests potential as a candidate marker that warrants further investigation.

Our study also revealed that the expression levels of ssc-miR-223, ssc-miR-202-3p, and ssc-miR-205 were significantly reduced in the Passed SP-EV group, as determined by FDR analysis. MiR-223 plays a critical role in immune cell differentiation, inflammation suppression, and platelet adhesion regulation [53]. Dysregulation of miR-223 has been associated with obesity, inflammatory disorders, cancer, autoimmune diseases, and reproductive dysfunction [54,55,56]. For example, elevated miR-223 expression has been observed in cows and horses with endometritis [57,58], while a study by Dominguez et al. [59] revealed an upregulation of miR-223 in patients with ectopic pregnancy. Additionally, a previous study showed that the presence of miR-223-3p in seminal plasma exosomes, along with miR-142, could serve as indicators of prostate cancer diagnosis [60]. Both miRNAs were downregulated in Passed SP-EVs in the current study, suggesting that their altered expression may contribute to reduced semen quality and reproductive disorders.

MiR-202-3p has been identified in the male reproductive system, where it is highly expressed in the testis and plays roles in inducing apoptosis and inhibiting tumor proliferation [61]. Notably, miR-202-3p maintains spermatogonial stem cells by negatively regulating the cell cycle and RNA-binding proteins [62]. In extracellular vesicles, Sun et al. [63] reported that ssc-miR-202-3p was upregulated in seminal plasma exosomes from semen containing spermatozoa with cytoplasmic droplets, an indicator of sperm immaturity. Conversely, Wainstein et al. [64] found that miR-202-3p was significantly downregulated in the seminal plasma of men with azoospermia. Furthermore, miR-205 plays an important role in regulating sperm maturation, motility, and survival by modulating the expression of the FoxO1 gene, which is localized in spermatogonial stem cells [65]. However, qPCR results from our study confirmed its downregulation in Passed SP-EVs. A similar trend was previously observed in boar SP-EV miRNAs during the winter season (log2FC = −1.418) [33], suggesting that miR-205 is a potential molecular marker of seasonal male infertility.

Among the five most abundant miRNAs in both SP-EV groups were ssc-miR-10b, ssc-miR-10a-5p, ssc-miR-200b, ssc-let-7a, and ssc-miR-26a. MiR-10a-5p is highly expressed in spermatogonia [66] and its abundance in SP-EVs suggests a potential testicular origin and a role in spermatogenesis. MiR-200b targets the porcine spermatogenesis-associated serine-rich 2-like (SPATS2L) gene, which influences litter size, indicating that SP-EVs may affect sperm function and embryo development [25,67].

Additionally, ssc-miR-26a regulates glucose metabolism by targeting the pyruvate dehydrogenase complex (PDHX), thereby influencing sperm viability [68]. MiR-26a also suppresses immune-related genes such as IL-6 and IL-7 [69], potentially affecting uterine receptivity. The let-7 family is among the most abundant miRNAs in boar, rabbit, and human SP-EVs [25,28,39] and is involved in inflammatory regulation and male germ cell differentiation [70,71]. Altogether, these findings indicate that SP-EV miRNAs participate in spermatogenesis, immune modulation, and embryo development.

Pathway enrichment analysis revealed that target genes of DEMs in both Passed and Failed SP-EVs were jointly enriched in PI3K-Akt, regulation of the actin cytoskeleton, and ErbB signaling pathways. These signaling pathways were reported to be enriched in high-motility semen exosomes and are crucial for regulating sperm motility in Landrace and Yorkshire boars [26]. The PI3K-Akt pathway is an intracellular signaling pathway that regulates boar sperm viability, as it includes two genes involved in cellular integrity: phosphoinositide 3-kinase (PI3K) and its target protein kinase B (PKB/AKT) [72]. It has been reported that the PI3K-Akt signaling pathway plays an important role in promoting spermatogonial proliferation and differentiation, enhancing sperm motility, regulating sperm autophagy, and testicular endocrine function [26,72,73,74]. In addition, the ErbB signaling pathway regulates gene expression in response to growth factors and was also enriched in high-semen-quality boar and duck SP-EVs, suggesting their regulatory role in sperm function [26,75].

Collectively, these results support the concept that seminal plasma EV-associated miRNAs act as key modulators of the sperm microenvironment and reflect physiological responses to seasonal heat stress. While most studies have focused on profiling SP-EV miRNAs under conditions such as age differences, cryopreservation, or overall semen quality, our study is novel in that it examines the potential role of SP-EV miRNAs in seasonal infertility, a major problem in swine reproduction. It also links EV miRNAs to mechanisms of seasonal sperm dysfunction, a connection that has not been well characterized previously. Consequently, this study demonstrates that EV miRNAs are altered by environmental conditions, such as heat, and may serve as season-specific signatures to predict summer infertility in boars and to identify potential mitigation interventions. However, further studies across additional commercial pig breeds and into the functional effects of SP-EVs on female reproductive physiology are required to fully elucidate their biological significance.

## 4. Materials and Methods

### 4.1. Semen Collection

Semen ejaculates were collected from 83 Duroc breeding boars aged 1.5 to 2 years at Prestage Farms, a commercial boar stud (West Point, MS, USA), which were housed in individual pens under similar management and controlled environmental conditions, with access to feed and water ad libitum. Trained farm personnel used the gloved-hand technique to collect semen samples over an eight-week period during the summer months (July–September), with two collections per week. On average, the barn temperature, ambient temperature, and humidity were 26 °C, 28 °C, and 76%, respectively. Semen samples were chilled and transported to our laboratory at Mississippi State University (Department of Animal and Dairy Sciences) within one hour for subsequent analyses. The study did not involve vertebrates because semen samples were collected at the board stud following standard procedures, with any excess donated for research (one collection per boar). Consequently, this work was exempt from review by the Mississippi State University Institutional Animal Care and Use Committee.

### 4.2. Semen Quality Evaluation

Boar semen diluted in phosphate-buffered saline (PBS) was incubated for 15 min in a 37 °C water bath. Two microliters were then loaded onto pre-warmed, multi-chamber microscope slides (Standard Count 4-Chamber Slide Leja^®^, 20 μm, Nieuw Vennep, The Netherlands). Sperm motility, progressive motility, normal morphology, and velocity parameters, including straight-line velocity (VSL), curvilinear velocity (VCL), and average path velocity (VAP), were assessed using a computer-assisted sperm analyzer (CEROS II, IMV Technologies, Brooklyn Park, MN, USA) under a phase-contrast microscope (CX-41, Olympus, Tokyo, Japan). The system was set to operate at 60 Hz (60 frames per second). Motility was categorized as VAP > 45 μm/s and VSL > 5 μm/s, with slow-moving cells defined as VAP < 20 μm/s and VSL < 5 μm/s. Progressive sperm were defined as those with VAP > 45 μm/s and straightness > 45%. Measurements were taken at 1.89× magnification with the stage temperature maintained at 37 °C. A total of 1500 sperm cells, randomly selected from five fields, were analyzed per sample to calculate total motility. Samples were classified as Passed (or high-quality: *n* = 38) if they contained ≥70% normal morphology and motile spermatozoa, whereas semen with <70% normal morphology and motile spermatozoa was classified as Failed (or low-quality: *n* = 45). Classification was determined immediately on-site after collection using sperm analysis cutoff criteria (Figure 1). Semen samples with extreme total motility and normal morphology values were selected from the 83 semen samples based on mean ± 2 SD, and those with Passed (*n* = 5) and Failed (*n* = 5) were used for EV isolation and miRNA sequencing (Figure 1).

### 4.3. Isolation of Seminal Extracellular Vesicles (SP-EVs)

The semen samples were centrifuged at 800× *g* for 20 min at 4 °C to remove sperm cells. The resulting supernatant was transferred to sterile tubes and then subjected to sequential centrifugation at 2000× *g* for 20 min at 4 °C, followed by 16,000× *g* for 1 h at 4 °C. The final supernatant was ultracentrifuged at 120,000× *g* for 70 min at 4 °C to pellet the EVs. The resulting pellet was resuspended in 5 mL filtered sterile PBS and washed twice by ultracentrifugation at 120,000× *g* for 70 min at 4 °C. The purified EV pellets were then resuspended in 100 µL of PBS and stored at −80 °C for further analysis.

### 4.4. Characterization of SP-EVs

#### 4.4.1. Nanoparticle Tracking Analysis (NTA)

The particle size and concentration of isolated boar SP-EVs were analyzed using the ZetaView^®^ QUATT Particle Size Analyzer (Particle Metrix, Holly Springs, NC, USA), at 488 nm and equipped with a fixed cell assembly. The system was calibrated and aligned using a 1:250,000 dilution of 100 nm polystyrene standard particles in aqueous suspension (Particle Metrix, Cat. 110-0020) for auto alignment and focus optimization. Prior to measurement, all samples were equilibrated to room temperature for 30 min. For each semen group, three frozen-thawed SP-EVs samples were diluted 1:2000 with sterile distilled water, and 1 mL was loaded into the analyzer. All measurements were performed under standardized conditions: room temperature, pH 7.0, sensitivity set to 65, and shutter speed set to 100. Measurements were taken at 11 positions per replicate (3 samples per group), with each sample analyzed in triplicate. Following video recordings, EV particle size and concentration were analyzed using ZetaView software (version 8.06.01 SP1). Outlier positions were automatically excluded by the software. The system was rinsed with 0.22 µm-filtered 1× PBS prior to each new measurement to prevent cross-contamination. SP-EV concentrations, accounting for the dilution factor, and average sizes were expressed as the number of SP-EV per microliter (particles/µL) and as nanometers (nm), respectively.

#### 4.4.2. Western Blot Analysis

Protein concentrations of Passed and Failed SP-EVs were quantified using the Micro BCA™ Protein Assay Kit (Cat. 23235, Thermo Fisher Scientific, Waltham, MA, USA) according to the manufacturer’s instructions. SP-EVs were then lysed with RIPA lysis buffer system (Cat. No. sc-24948, Santa Cruz Biotechnology Inc., Dallas, TX, USA) by centrifuging at 12,000× *g* for 30 min at 4 °C and quantified using the Bicinchoninic acid assay (Micro BCA Protein Assay Kit, Cat. No. 23235, Waltham, MA, USA). Approximately 30 µg of protein was separated on a 4–12% gradient SDS-PAGE polyacrylamide gel and transferred onto PVDF membranes. The membranes were then blocked with blocking buffer (WesternBreeze Chromogenic Immunodetection System, Cat. No. WB7103, Thermo Fisher Scientific, Carlsbad, CA, USA) for 1 h at room temperature and subsequently incubated overnight at 4 °C with primary mouse monoclonal antibodies against CD9 (1:200, Cat. No. sc-13118, Santa Cruz, Dallas, TX, USA), CD63 (1:200, Cat. No. sc-5275, Santa Cruz, Dallas, TX, USA), CD81 (1:200, Cat. No. sc-166029, Santa Cruz, Dallas, TX, USA), and β-Tubulin (1:200, Cat. No. sc-58886, Santa Cruz, Dallas, TX, USA). After washing three times with antibody wash, the membranes were incubated with an alkaline-phosphate conjugated anti-mouse secondary antibody (1:20,000, Cat. No. 46-7006, Thermo Fisher Scientific, Carlsbad, CA, USA) for 1 h at room temperature. Immunodetected proteins were visualized using a chromogenic substrate, and protein bands were analyzed accordingly.

### 4.5. Total RNA Extraction and Sequencing

Total RNA from SP-EVs was isolated using the Exosomal RNA Isolation Mini Kit (Norgen Biotek Corp., Thorold, ON, Canada) according to the manufacturer’s instructions. RNA concentration and purity were assessed using a NanoDrop One spectrophotometer (Thermo Scientific, Waltham, MA, USA), and RNA integrity was evaluated with Agilent 2100 Bioanalyzer (Agilent Biotechnologies, Santa Clara, CA, USA). Samples were then submitted to Norgen Biotek Corp. for small RNA sequencing on the Illumina^®^ NextSeq 550 platform (Illumina, Inc., San Diego, CA, USA). Sequencing libraries were prepared using the Norgen Biotek Small RNA Library Prep Kit (Cat. 63620, Norgen Biotek Corp., Thorold, ON, Canada).

#### 4.5.1. Small RNA Sequencing Data Analysis

The workflow was performed as previously described by Aparicio-Puerta et al. [76]. Clean reads were obtained from raw sequencing data and mapped to the *Sus scrofa* reference genome (Ensembl Release 89, version 11.1) using RNAcentral. The quality of raw FASTQ files was assessed with FASTQC (version 0.11.4; http://www.bioinformatics.babraham.ac.uk/projects/fastqc/, accessed on 29 July 2022). Reads were trimmed based on quality (Q-score > 30) and read length. High-quality reads were then aligned to the pig genome and annotated against porcine precursor and mature miRNAs using miRBase (release 20, https://www.mirbase.org/, accessed on 29 July 2022). Secondary structure prediction was performed using TargetScan (https://www.targetscan.org/, accessed on 10 March 2025) and miRWalk (http://mirwalk.umm.uni-heidelberg.de/, accessed on 10 March 2025). Raw expression data were normalized with the trimmed mean of M-values (TMM) method, and results were reported as TMM-adjusted counts per million (CPM), accounting for both log-fold changes and absolute gene-wise expression differences across samples. A minimum CPM threshold of 5 was applied to filter miRNAs. All analyses were conducted using TMM-normalized expression values in R software (version 3.6.3) with the EdgeR statistical package (version 3.24.0). MiRNAs were considered differentially expressed (DEMs) based on a log2 fold change ≥1 or ≤−1, a *p*-value < 0.05, and an average CPM > 5. Hierarchical clustering of all DE miRNAs was performed using the ComplexHeatmap package (version 1.20.0).

#### 4.5.2. Target Gene Prediction and Bioinformatic Analysis

The human miRNA homologs for the DE boar SP-EV miRNAs were identified using the miRBase database (https://www.mirbase.org/, accessed on 6 March 2025). These were then used for target gene prediction using the miRWalk database (http://mirwalk.umm.uni-heidelberg.de/, accessed on 10 March 2025) [77]. Within miRWalk, validated target genes from miRTarBase (version 7.0) and commonly predicted target genes by miRDB (release 5.0) and TargetScan (version 7.1, https://www.targetscan.org/, accessed on 10 March 2025) were selected for gene ontological (GO) classification and Kyoto Encyclopedia of Genes and Genomes (KEGG) pathway enrichment analyses using the DAVID bioinformatics web tool (https://davidbioinformatics.nih.gov/; accessed on 12 March 2025). Pathways with a *p* < 0.05 were considered significant. Venn diagrams comparing lists of miRNAs were created using the online tool Venny 2.1.0 BioinfoGP (https://bioinfogp.cnb.csic.es/tools/venny/index.html, accessed on 20 March 2025).

#### 4.5.3. Validation of miRNA Expression by Quantitative Real-Time PCR

Total RNA was isolated from Passed and Failed SP-EVs using the miRNeasy Micro Kit (Cat. No. 217084, QIAGEN, Redwood City, CA, USA) according to the manufacturer’s instructions. Complementary DNA (cDNA) was synthesized using the TaqMan™ Advanced miRNA cDNA Synthesis Kit (Cat. No. A28007, Applied Biosystems, Waltham, MA, USA) following the manufacturer’s protocol. Thermal cycling was performed on a QuantStudio 3 Thermal Cycler (Applied Biosystems, Waltham, MA, USA), and the synthesized cDNA was stored at −20 °C until further analysis. Quantitative real-time PCR (qPCR) was carried out using the TaqMan Fast Advanced Master Mix (Cat. No. 4444556, Applied Biosystems) and pre-designed TaqMan™ Advanced miRNA Assays (Cat. A25576, Applied Biosystems, Waltham, MA, USA) for human miRNAs that have been validated in porcine species, including hsa-miR-205, hsa-miR-802, hsa-miR-7-1-3p, hsa-let-7a, and hsa-miR-26a as an endogenous control. Mir-26a was utilized to normalize miRNA expression because of its stability and was also among the top 10 most abundant miRNAs in both SP-EV groups. Relative expression levels of miRNAs were calculated using the comparative threshold cycle method (2^−ΔΔ^Ct), with normalization to internal controls.

### 4.6. Statistical Analysis

Semen quality data were analyzed using RStudio statistical software version 4.5.2 (Boston, MA, USA). Data normality was assessed using the Shapiro–Wilk test. Group comparisons between Passed and Failed semen samples were conducted using the Wilcoxon rank sum test. Differences were considered statistically significant at *p* < 0.05. Data were expressed as mean ± standard error of the mean (SEM), and graphs were plotted using the GraphPad Prism (version 11.0.0).

## 5. Conclusions

In summary, this study identified unique DE miRNAs in boar SP-EVs involved in several sperm biological processes, including sperm maturation, spermatogenesis, and sperm quality. MiR-205 was also identified as a promising candidate biomarker of male infertility during seasonal changes. Thus, the presence of this miRNA in SP-EVs may serve as a potential indicator for predicting boar semen quality and as a candidate for therapies targeting environmental stress in commercial swine production systems.

## Figures and Tables

**Table 1:** List of differentially expressed (DE) miRNAs between Passed and Failed SP-EVs.

miRNAs	log2FC	Fold Change (FC)	*p*-Value	FDR	Up/Downregulated (In Passed SP-EVs)
ssc-miR-223	−3.98	0.06	6.11 × 10^−6^	2.01 × 10^−3^	Downregulated
ssc-miR-202-3p	−2.03	0.24	1.04 × 10^−5^	2.01 × 10^−3^	Downregulated
ssc-miR-205	−1.46	0.36	4.89 × 10^−5^	6.29 × 10^−3^	Downregulated
ssc-miR-1224	−1.80	0.29	1.51 × 10^−3^	0.15	Downregulated
ssc-miR-194a-5p	−1.59	0.33	2.59 × 10^−3^	0.16	Downregulated
ssc-miR-484	−1.65	0.32	3.27 × 10^−3^	0.16	Downregulated
ssc-miR-503	−1.17	0.44	3.34 × 10^−3^	0.16	Downregulated
ssc-miR-192	−1.41	0.38	3.38 × 10^−3^	0.16	Downregulated
ssc-miR-142-5p	−1.24	0.42	3.62 × 10^−3^	0.16	Downregulated
ssc-miR-1468	−1.07	0.48	7.04 × 10^−3^	0.25	Downregulated
ssc-miR-9793-5p	−2.11	0.23	7.23 × 10^−3^	0.25	Downregulated
ssc-miR-124a	−2.49	0.18	9.46 × 10^−3^	0.29	Downregulated
ssc-miR-127	−1.28	0.41	9.88 × 10^−3^	0.29	Downregulated
ssc-miR-802	−1.10	0.47	1.32 × 10^−2^	0.36	Downregulated
ssc-miR-9846-3p	−1.49	0.36	1.39 × 10^−2^	0.36	Downregulated
ssc-miR-9802-3p	−2.38	0.19	1.75 × 10^−2^	0.42	Downregulated
ssc-miR-202-5p	−0.99	0.50	2.74 × 10^−2^	0.60	Downregulated
ssc-miR-450c-5p	−0.90	0.54	2.80 × 10^−2^	0.60	Downregulated
ssc-miR-424-3p	−1.02	0.49	3.21 × 10^−2^	0.63	Downregulated
ssc-miR-551a	1.02	2.03	3.26 × 10^−2^	0.63	Upregulated
ssc-miR-9824-5p	−1.88	0.27	3.59 × 10^−2^	0.63	Downregulated
ssc-miR-504	−1.31	0.40	3.63 × 10^−2^	0.63	Downregulated
ssc-miR-9852-3p	−1.14	0.45	4.07 × 10^−2^	0.63	Downregulated
ssc-miR-671-5p	−1.31	0.40	4.19 × 10^−2^	0.63	Downregulated
ssc-miR-146a-5p	−0.54	0.69	4.55 × 10^−2^	0.63	Downregulated
ssc-miR-450a	−0.79	0.58	4.65 × 10^−2^	0.63	Downregulated
ssc-miR-196a	−0.67	0.63	4.69 × 10^−2^	0.63	Downregulated
ssc-miR-7-3p	1.09	2.13	4.78 × 10^−2^	0.63	Upregulated
ssc-miR-9784-5p	−2.67	0.16	4.82 × 10^−2^	0.63	Downregulated
ssc-miR-758	−1.67	0.31	4.91 × 10^−2^	0.63	Downregulated

## Data Availability

All data are provided in the manuscript.

## Abbreviations

The following abbreviations are used in this manuscript:

AI	Artificial insemination
CASA	Computer-assisted sperm analysis
CPM	Counts per million
DAVID	Database for annotation, visualization, and integrated discovery
DE	Differentially expressed
DEMs	Differentially expressed miRNAs
DNA	Deoxyribonucleic acid
EGFR	Endothelial growth factor receptor
EVs	Extracellular vesicles
FC	Fold change
FDR	False discovery rate
GO	Gene ontology
IL	Interleukin
KEGG	Kyoto encyclopedia of genes and genomes
MAPK	Mitogen-activated protein kinase
MISEV	Minimal information for studies of extracellular vesicles
NTA	Nanoparticle tracking analysis
PCR	Polymerase chain reaction
PDHX	Pyruvate dehydrogenase complex
PI3K	Phosphoinositide 3-kinase
PVDF	Polyvinylidene fluoride
RNA	Ribonucleic acid
RT-qPCR	Real-time quantitative polymerase chain reaction
SD	Standard deviation
SDS-PAGE	Sodium dodecyl sulfate–polyacrylamide gel electrophoresis
SEM	Standard error of the mean
SP	Seminal plasma
SPATS2L	Spermatogenesis-associated serine-rich 2-like
VAP	Average path velocity
VCL	Curvilinear velocity
VSL	Straight-line velocity
